# Mobile Phone Applications for the Care and Prevention of HIV and Other Sexually Transmitted Diseases: A Review

**DOI:** 10.2196/jmir.2301

**Published:** 2013-01-04

**Authors:** Kathryn E Muessig, Emily C Pike, Sara LeGrand, Lisa B Hightow-Weidman

**Affiliations:** ^1^Department of MedicineThe University of North Carolina at Chapel HillChapel Hill, NCUnited States; ^2^Center for Health Policy and Inequalities ResearchDuke UniversityDurham, NCUnited States

**Keywords:** HIV, technology, mobile phone applications

## Abstract

**Background:**

Mobile phone applications (apps) provide a new platform for delivering tailored human immunodeficiency virus (HIV) and sexually transmitted disease (STD) prevention and care.

**Objective:**

To identify and evaluate currently available mobile phone apps related to the prevention and care of HIV and other STDs.

**Methods:**

We searched the Apple iTunes and Android Google Play stores for HIV/STD-related apps, excluding apps that exclusively targeted industry, providers, and researchers. Each eligible app was downloaded, tested, and assessed for user ratings and functionality as well as 6 broad content areas of HIV prevention and care: HIV/STD disease knowledge, risk reduction/safer sex, condom promotion, HIV/STD testing information, resources for HIV-positive persons, and focus on key populations.

**Results:**

Search queries up to May 2012 identified 1937 apps. Of these, 55 unique apps met the inclusion criteria (12 for Android, 29 for iPhone, and 14 for both platforms). Among these apps, 71% provided disease information about HIV/STDs, 36% provided HIV/STD testing information or resources, 29% included information about condom use or assistance locating condoms, and 24% promoted safer sex. Only 6 apps (11%) covered all 4 of these prevention areas. Eight apps (15%) provided tools or resources specifically for HIV/STD positive persons. Ten apps included information for a range of sexual orientations, 9 apps appeared to be designed for racially/ethnically diverse audiences, and 15 apps featured interactive components. Apps were infrequently downloaded (median 100-500 downloads) and not highly rated (average customer rating 3.7 out of 5 stars).

**Conclusions:**

Most available HIV/STD apps have failed to attract user attention and positive reviews. Public health practitioners should work with app developers to incorporate elements of evidence-based interventions for risk reduction and improve app inclusiveness and interactivity.

## Introduction

Mobile phone health interventions are increasingly being used for the prevention and care of human immunodeficiency virus (HIV) and other sexually transmitted diseases (STDs) [1-3]. These initiatives have been designed to promote prevention messages [4], facilitate test result notification [5,6], improve HIV medication adherence, and increase adherence to clinic appointments [7-12]. Although phone-based interventions have typically used the voice or text-based Short Message Service (SMS) features of mobile phones [1,3,13], the increasing popularity of smartphones and smartphone applications (apps) [14,15] has greatly expanded the possibilities for phone-based HIV/STD interventions [13]. These interventions are critical for reversing the HIV epidemic; 34.2 million people worldwide are living with HIV and 2.5 million people became newly infected in 2011 alone [16].

Smartphones have revolutionized mobile communication markets by offering enhanced mobile phones featuring improved Internet access and the capacity to perform more advanced computer functions. Consumer research from June 2012 estimates that 54.9% of mobile phone subscribers in the United States own a smartphone, with phones running on Apple’s iPhone operating systems (iOS) and Android’s operating systems (Android OS) representing over 86% of this market [15]. Smartphone apps are downloadable programs that run on the smartphone’s OS, which may include Web-based features. As of March 2012, Apple’s iTunes apps had received over 25 billion downloads [14] and the Android Google Play Store exceeded 15 billion app downloads in May 2012 [17]. These numbers continue to rise at over 1 billion app downloads per month for each store [17].

Many of these apps are health related. A search on August 1, 2012, for apps categorized as “health & fitness” and “medical” yielded 13,479 apps available for iPhone consumers [18] and 15,891 apps available for Android consumers [19]. This widespread use of apps provides a promising new platform for delivering tailored HIV prevention messages and interactive care services. But are public health practitioners utilizing this new opportunity? In this paper, we review the characteristics and content of HIV- and STD-related apps that are available through the two primary online app providers: the Apple iTunes Store and the Android Google Play Store. This review describes the current landscape and content of HIV/STD apps, assesses utilization and acceptability of these apps, and provides recommendations to guide the design and development of future apps.

## Methods

### Search and Screening Strategy

On May 1 to 3, 2012, the following terms were used to search the Apple iTunes Store and the Android Google Play Store: HIV, human immunodeficiency virus, acquired immune deficiency syndrome, sexually transmitted diseases, STD, sexually transmitted infections, STI, sexual health, safe sex, and condom. An app was excluded if it did not include HIV/STD content; exclusively targeted industry, health care, research, or medical professionals; or was not available in English. Two researchers (KEM and ECP) searched each store and compiled lists of all identified apps. App titles and descriptions were screened for relevance and lists of apps to download were created. Lists were compared and differences resolved by a third member of the study team (LBH). Any eligible app that was identified in only one store was searched for by name in the other store to confirm its exclusive availability.

### Data Extraction and App Assessment

The following data was extracted for each eligible app on May 1 to 3, 2012: name, platform (iPhone, Android, or both), category as defined by site (eg, medical, education, health and fitness, lifestyle), description of app content as provided by site, price, user star rating, number of customer downloads (available for Android only), number of customer ratings (available for iPhone only), and the date the app was last updated by the developer. For apps that were available in both the Google Play and iTunes stores, we recorded separate price and user rating information for each store. From May 7 to 24, 2012, we downloaded each eligible app (both free and fee-based) and tested all features and functions. If an app had both a free and fee-based version, only the free version was downloaded and assessed.

To evaluate the apps we identified 6 broad content areas for advancing the prevention and treatment of HIV based on the World Health Organization’s Global Health Sector Strategy on HIV/AIDS 2011-2015 [20] and the National AIDS Strategy for the United States [21]. These content areas include HIV/STD knowledge and awareness, behavior change/risk reduction/safer sex promotion, condom use/promotion, HIV/STD testing, resources and linkage to care for HIV/STD-positive persons, and intensified or focused efforts for key communities/populations. Each app was assessed for inclusion of the 5 content areas, operationalized as:

1. Does the app provide information about HIV or other STDs?

2. Does the app provide information or descriptions about ways to reduce the risk of sexually transmitting or acquiring HIV/STDs?

3. Does the app provide information about how to use or obtain male or female condoms?

4. Does the app promote or provide information or resources about HIV/STD testing?

5. Does the app provide resources specifically for HIV/STD-positive persons? (Features and resources provided through apps for HIV/STD-positive persons were tested and described.)

If the app also covered other content areas, these were listed and described. These descriptions were then compiled and grouped, resulting in the following additional 5 content categories: drug and alcohol risk, relationships, HIV/STD news, HIV/STD stigma, and an HIV/STD status verification service.

To assess whether the app fulfilled the criteria of intensified or focused efforts for key communities or populations, we reviewed the text and images used in each app. Key populations for HIV prevention vary depending on the local epidemic context. Thus, this assessment included an open-ended description of text and images used and a subjective assessment of whether the app appeared to be inclusive or tailored. For example, if an app included images of black men or women among a variety of racial and ethnic images, this app would be characterized as “inclusive.” If an app explicitly or exclusively addressed black men or women, this app would be considered “tailored.” Finally, we assessed whether each app included any interactive component, such as a game, quiz, diary, or goal tracker.

## Results

### Search and Screening

**Figure 1:**
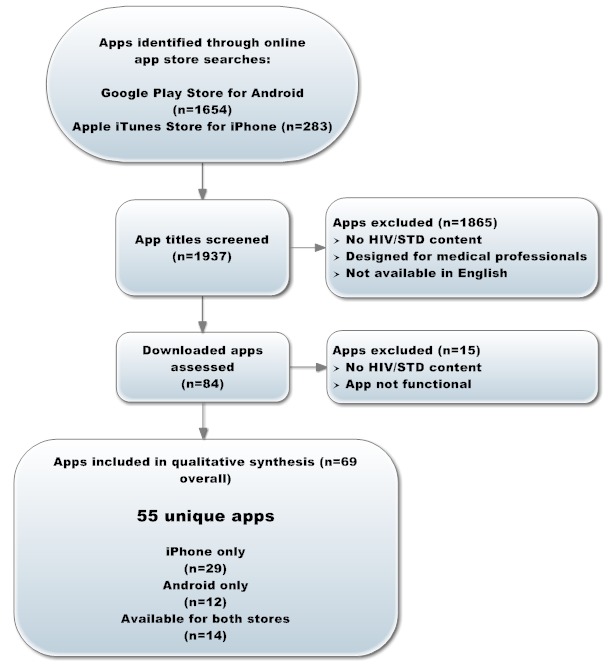
Search and screening process for HIV/STD-related apps.

Search queries identified 1937 apps. After screening the app titles and the site-provided app descriptions, 84 eligible apps were downloaded for full review (Figure 1). A further 15 apps were then excluded because they did not include HIV/STD-related content or were not functional (list available upon request). A total of 69 apps met the final inclusion criteria: 43 from the Apple iTunes Store and 26 from Android’s Google Play Store. Of these, 55 apps were unique with 14 apps available in both stores (29 iTunes only, 12 Google Play only, and 14 available in both stores).

### Descriptive Characteristics

Table 1 provides a summary of app characteristics and Table 2 lists the name, details, and primary content areas of each eligible app. Approximately half (27/55, 49%) of the apps had been updated within the past year (May 2011 to May 2012). Seventy-one percent (39/55) of apps provided information about HIV/STDs and 24% (13/55) provided information about reducing sexual transmission risk. Twenty-nine percent (16/55) of apps included instructions for condom use or assistance locating condoms and 36% (20/55) included information about HIV/STD testing or resources for finding testing centers. Only 6 apps covered all 4 of these broad areas of HIV/STD prevention (knowledge, risk reduction, condom promotion, and testing): Action for AIDS [22], Pos or Not [23], STD411 [24], STDiQ [25], Safe Sex Tips [26], and Sexual Health Guide [27]. In addition, one app supported a third-party service that provided an electronic certificate verifying negative HIV/STD test results (Chec-Mate) [28]. Five (9%) apps addressed (noninjection) drug or alcohol use and HIV/STD risk. A small number of apps discussed HIV/STDs in the context of relationships (n=5), provided links to online news stories featuring HIV/STDs (n=4), or addressed HIV stigma (n=1).

**Table 1:** Summary of mobile phone applications (apps) for HIV/STD prevention and care (N=55).

App characteristic		n	%
Phone platform			
	iPhone	29	52.7
	Android	12	21.8
	Both	14	25.5
App price^a^ (US $)			
	Free	46	66.7
	0.99	14	20.3
	1.00-9.99	9	13.0
Customer app rating^a^			
	Unrated^b^	31	44.9
	0.0-3.9 stars	16	23.1
	4.0-5.0 stars	22	31.8
Number of app downloads^c^			
	0-100	12	46.2
	101-500	3	11.5
	501-999	1	3.8
	1000-9999	5	19.2
	> 10,000	5	19.2
Updated since May 2011			
	Yes	27	49.1
	No	28	50.9
App category			
	Brain and puzzle	1	1.8
	Casual	1	1.8
	Education	6	10.9
	Entertainment	1	1.8
	Games	1	1.8
	Health and fitness	20	36.4
	Lifestyle	10	18.2
	Medical	13	23.6
	Social networking	1	1.8
	Utilities	1	1.8
Focused for particular populations^d^			
	Geographically focused	13	23.6
	Inclusive of black or Latino users	9	16.4
	Tailored for black or Latino users	2	3.6
	Information for MSM	7	12.7
	Tailored for MSM	2	3.6
HIV/STD-focused content/features^d^			
	HIV/STD information	39	70.9
	HIV/STD testing	20	36.4
	Condom use, condom locator	16	29.1
	Game/quiz/risk assessment	15	27.3
	Safer sex	13	23.6
	Tools for HIV/STD-positive persons	8	14.5
	Drugs, alcohol (HIV/STD risk)	6	10.9
	Relationships	5	9.1
	HIV/STD news	4	7.3
	Stigma	1	1.8
	HIV/STD status verification service	1	1.8

^a^ Calculated based on 69 total apps because Google Play and iTunes occasionally charged different prices for the same app and received different customer ratings.

^b^ Apps that have not received any user ratings.

^c^ Information on number of app downloads is only available for apps sold in the Android Google Play Store (n=26).

^d^ Each app may be categorized with multiple content areas. Percentages were calculated as percent of unique apps (n=55) that include this content area. HIV: human immunodeficiency virus; STD: sexually transmitted disease; MSM: men who have sex with men.

**Table 2:** Characteristics and content of mobile phone HIV/STD applications (apps).

App name		Price (US $)	Rating^a^	Down­loads^b^	Targeted population^c^	App focus					
						Know­ledge	Safer sex	Con­doms^d^	Testing^e^	HIV/ STD+^f^	Other
**iPhone apps**											
	Action For AIDS	0	—	—	Singapore	Y	Y	Y	Y		
	AIDSinfo HIV/AIDS Glossary	0	4.5	—	Inclusive: Spanish	Y					
	aidsmap news	0	—	—	NT	Y					News
	amfAR TestingDay	0	—	—	NT	Y			Y		Quiz
	Chec-Mate	1.99^g^	—	—	NT				Y		HIV/STD status verification service
	Condom Pro	0	—	—	NT			Y			Game
	Condom Truth	0	—	—	NT			Y			
	Hiv & Aids Guide	0.99	—	—	Anal sex	Y	Y	Y			
	HIV and Your Heart	0	5	—	Inclusive: black women	Y				Y	
	HIV Study (AIDS disease)	0.99	—	—	NT	Y			Y		
	i*********e-info	0	2.5	—	NT	Y					
	iCondom	0	5	—	NT			Y			
	iCondom Philly	0	4.5	—	Philadelphia			Y			
	It’s Your Shout	0	—	—	England				Y		Quiz
	LoveSmarts	0.99	—	—	NT	Y	Y				Game
	MASTDinfo	0	—	—	Massachusetts; tailored: MSM	Y			Y		
	Men’s Sexual Health	4.99	3	—	Inclusive: black men	Y					
	My First Time	0	3	—	China/ Singapore	Y			Y		
	Pos or Not	0	4	—	Inclusive: racial/ethnic minorities	Y	Y	Y	Y		Stigma, quiz/game
	PozTracker	3.99	—	—	NT	Y				Y	
	Safe Sex	0	4	—	NT		Y				
	Safesex Guide	1.99	—	—	Denmark	Y	Y				Alcohol, relationships, quiz
	Sexual Health News Reader	0.99	—	—	NT	Y					News
	STD Guide	1.99	3	—	NT	Y			Y		
	STD411	0	2	—	San Francisco; tailored: gay, bi, trans	Y	Y	Y	Y		Risk assessment
	STDiQ	0	4	—	Tailored: black, Latino	Y	Y	Y	Y		Alcohol/drugs, risk assessment, relationships
	Stop AIDS	0	4	—	Anal sex	Y					
	TalkPositive	0	—	—	NT					Y	
	TKNO	0	—	—	Inclusive: black men				Y		
**Android apps**											
	About HIV	0	4.5	++	NT	Y			Y		News
	Chlamydia	0	—	++	NT	Y			Y		
	Guide to STDs	5.00	1	+	NT	Y					
	HIV RISK Calculator	0	5	+	Inclusive: MSM, anal sex	Y			Y		Risk assessment
	Hook-ups: STD Rally	0	3.5	++++	NT	Y					Game
	Kenny Condom	0.99	—	+	Tailored: young African American men			Y	Y		
	Know Sexual Health	0	2.7	++++	NT	Y					Quiz
	Protection-Sex	0.99	—	+	England	Y	Y	Y			
	Safer Sex	0.99	—	+	England; inclusive: gay youth, anal sex	Y	Y	Y			
	Sex Detective	0.99	1.9	++++	Inclusive: anal sex	Y					Risk assessment
	Sex Guide	0.99	—	+	England; inclusive: gay youth	Y	Y				Drugs, alcohol, relationships
	STD Risk Calculator Lite	0	5	++	Inclusive: MSM, anal sex						Risk assessment
**Available on iPhone and Android**											
	AfterSex	0.99/0^h^	3.5/3.6	++++	Inclusive: ethnic Minorities	Y			Y		Risk assessment
	Big Night Out	0.99	—/—	+	England	Y		Y			Drugs, alcohol
	Birmingham AIDS Outreach (BAO)	0	5/5	+	Inclusive: MSM and males of color	Y			Y	Y	
	iStayHealthy	0	—/4.7	++	NT					Y	
	Patient Treatment Companion	0	—/—	+	NT					Y	
	NYC Condom finder	0	5/4	++++	New York City			Y			
	PositiveSingles	0	2/2	+++	NT					Y	
	Red Ribbon HIV/AIDS Manager	9.99/ 1.99^i^	—/4.7	+	NT					Y	
	Safe Sex Tips	0/1.14^j^	4/—	+	Inclusive: anal sex	Y	Y	Y	Y		Drugs, alcohol, relationships
	Sex Facts	0	3.5/4	+++++	Anal sex	Y					
	SeX Factor	0	—/2.2	++++	NT	Y					Game
	Sexual Health	0	3/4	+++	NT	Y					
	Sexual Health Guide	0	—/5	+++	Ireland; inclusive: anal sex	Y	Y	Y	Y		Drugs, alcohol, relationships, news
	STD Glossary	0.99	—/4	++	NT	Y					

^a^ —: not yet rated. For apps available for both iPhone and Android, ratings presented as iPhone/Android.

^b^ Download data was recorded on May 1-3, 2012, and was only available for Android Google Play store; —: not reported; +: 1-100 downloads; ++: 101-1000 downloads; +++: 1001-5000 downloads; ++++: 5001-50,000 downloads; +++++: 50,000-100,0000 downloads.

^c^ NT: not inclusive or tailored for specific geographic area or population(s); MSM: men who have sex with men.

^d^ Condom use and condom GPS locators.

^e^ Testing promotion and clinic locators.

^f^ Information, services, and/or care management tools for persons diagnosed with HIV or other sexually transmitted diseases (STDs).

^g^ For 1-year membership.

^h^ iPhone version $0.99.

^i^ iPhone version $9.99.

^j^ iPhone version free.

### Apps for Persons Diagnosed With HIV or Other STDs

Out of the 55 apps, 8 (15%) were developed for persons diagnosed with HIV or other STDs. These apps typically included interactive tools for the following areas: medication adherence tracking and reminders, medication interaction information, medical appointment calendars and reminders, doctor/clinic names and locations, symptoms/side effects trackers, and viral load/lymphocyte (CD4) cell count trackers (Table 3). One social networking app (PositiveSingles) [29] facilitated connections with other persons diagnosed with HIV or other STDs.

**Table 3:** Apps for HIV/STD-positive persons.

Name	Platform	Price (US $)	Features
Birmingham AIDS Outreach	iPhone, Android	Free	Connect to resources and support groups
HIV and Your Heart	iPhone	Free	Track CD4, viral load, weight, smoking, and other heart health measures
iStayHealthy	iPhone, Android	Free	Track CD4, viral load, medications, adherence, side effects, illnesses; alarm
Patient Treatment Companion	iPhone, Android	Free	Track CD4, viral load, medications, appointments, weight; alarm
PositiveSingles	iPhone, Android	Free	Connect to other positive persons
PozTracker	iPhone	3.99	Track CD4, viral load, medications, clinic locations, allergies, insurance; alarm
Red Ribbon HIV/AIDS Manager	iPhone, Android	9.99, 1.99	Record allergies, medications, CD4, viral load; alarm
Talk Positive	iPhone	Free	Track side effects, CD4, viral load, medications, appointments, allergies

**Figure 2:**
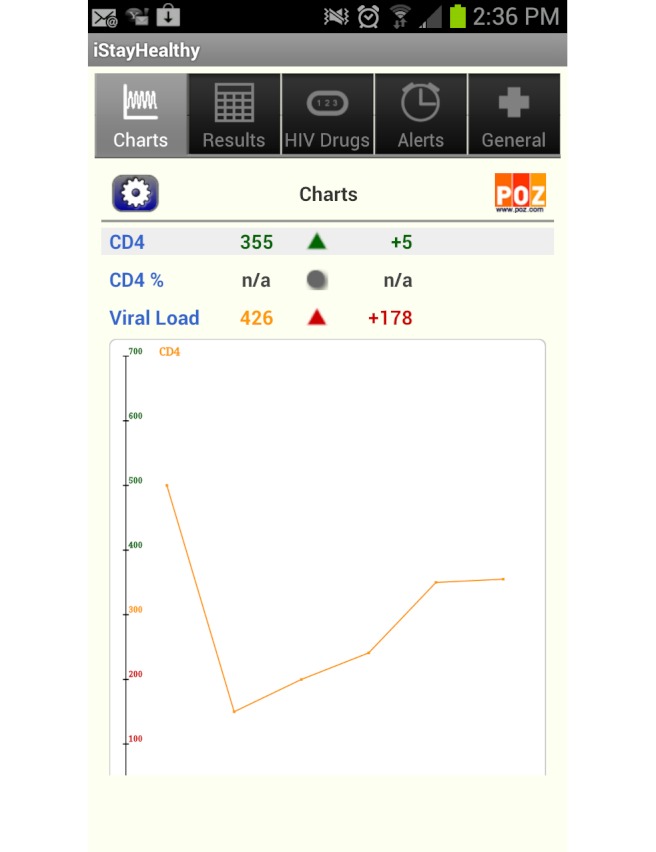
Screenshot of the iStayHealthy app’s user-generated CD4 cell count tracking. Android version shown on Samsung Galaxy S IV. Actual phone screen size: 9.6 × 5.5 cm. Reproduced with permission from creator, Dr Peter Schmidt.

**Figure 3:**
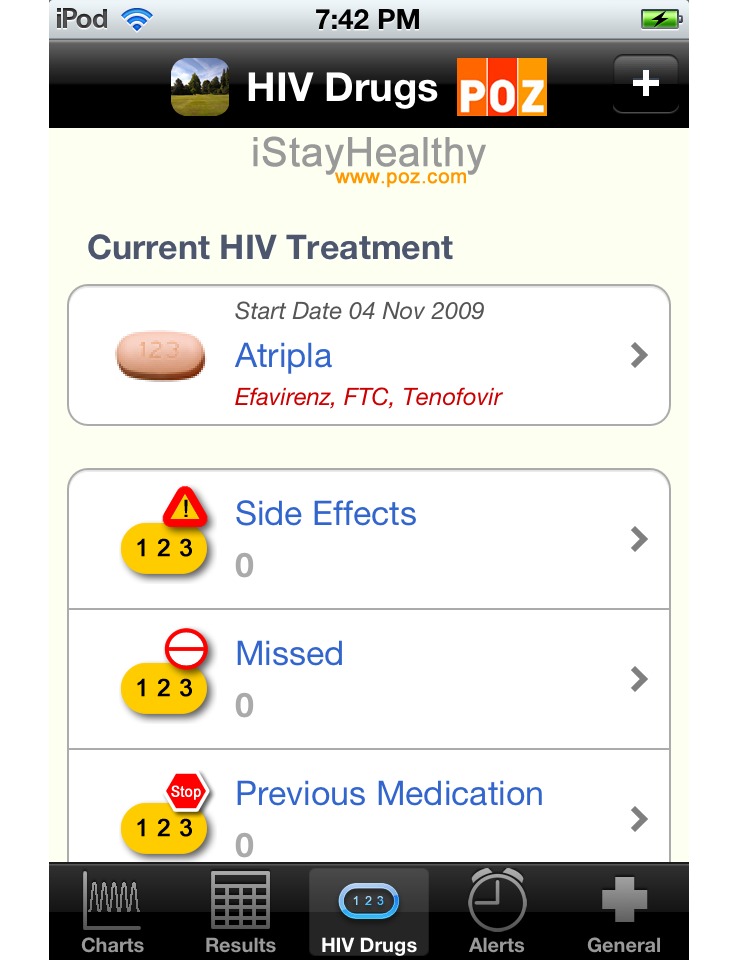
Screenshot of iStayHealthy app’s user-generated HIV medication list. iPhone version shown on Apple iPhone 4. Actual phone screen size: 6.8 × 4.5 cm. Reproduced with permission from creator, Dr Peter Schmidt.

The two highest user-rated apps for HIV/STD-positive persons were HIV and Your Heart [30] (5 stars) and iStayHealthy [31] for both the iPhone and Android (4.7 stars). The HIV and Your Heart [30] app included videos featuring providers, experts, and HIV-positive persons. This app also provided information about HIV and heart health, smoking cessation, diet and exercise, and a wellness checklist for tracking 9 health measures, including viral load and CD4 cell counts. The iStayHealthy [31] app provided a tracking and charting feature for viral load and CD4 cell counts (Figure 2), a component to record missed medication doses and medication side effects, a medication reminder alarm, and a place to record other medical history data, such as clinic locations and illness events. This app also included a photo library of HIV medication pill images that could be tailored to a daily medication schedule (Figure 3).

### Interactive App Components

**Figure 4:**
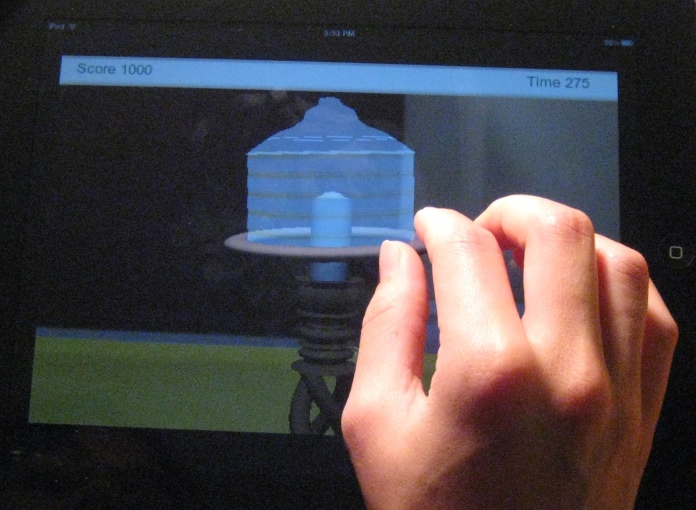
Screenshot of user placing a condom on a practice object in the interactive Condom Pro app. iPhone version shown on Apple iPad 2 for clarity. Reproduced with permission from Liz Sabatiuk, Social Media Manager, The National Campaign to Prevent Teen and Unplanned Pregnancy.

**Figure 5:**
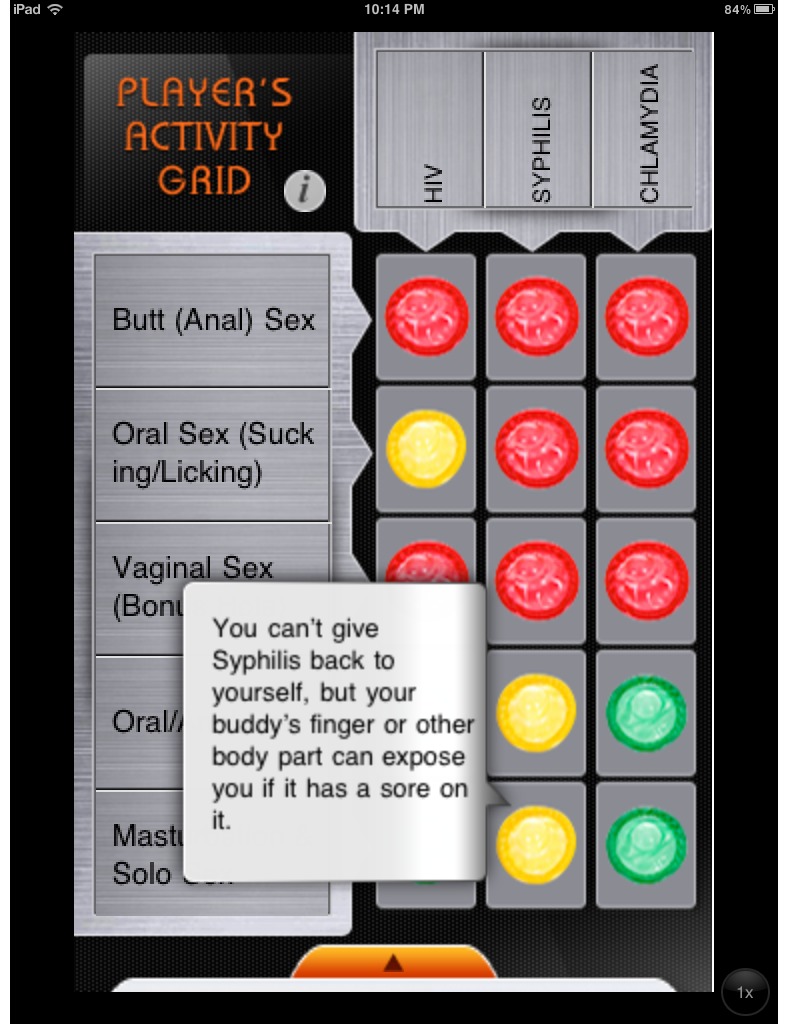
Screenshot of the MSM-tailored, interactive sexual behavior risk chart from STD411. iPhone version shown on Apple iPad 2 for clarity.

Fifteen apps (27%) that did not specifically target HIV-infected persons included an interactive component, such as a game/quiz (n=9) or a risk assessment activity (n=6). Here we highlight 4 examples of different types of interactive components (full descriptions of all interactive app components available upon request). In Condom Pro [32], players advance through 10 game levels by using the phone’s touchscreen to carefully “open” a condom, correctly place it over an object (Figure 4), and correctly remove it from the object. It’s Your Shout [33] asks the user a series of humorous personality questions and then matches the user’s profile with a superhero or celebrity’s profile. The final message with each quiz announces that, “Even superheroes can get Chlamydia. Be tested. Be sure.” The user is then given the option to order a home testing kit for chlamydia screening. The STD411 [24] app features an interactive chart for gay, bisexual, and transgender men where flashing colored condoms (red, yellow, and green) indicate the level of risk of different sexual activities for various STDs (Figure 5). As the user selects various colored condoms, diseases, or sexual activities, a brief risk explanation appears above the graphic. Finally, SeX Factor [34] includes a game in which the user moves across a game board by choosing and correctly answering an HIV/STD quiz question. Users who successfully cross the game board win a free chlamydia home test kit.

### App Ratings

Customer ratings and number of app downloads roughly measure an app’s acceptability and popularity. More than half (38/69, 55%) of the apps were customer rated yielding an average store rating of 3.7 out of 5 stars. In general, apps were infrequently downloaded (median 100-500 downloads) with only 11 apps exceeding 1000 downloads. Most (46/69, 67%) of the apps were free; the remainder ranged in price from US $0.99 to $9.99. Among apps that charged a fee, those charging over the median of US $0.99 all received fewer than 100 downloads.

The most popular app based on reported number of downloads was Sex Facts [35] that had a star rating of 4.0 and downloads between 500,000 and 1,000,000. This app featured a revolving index of sex-related factoids that could be shared with friends via social network sites (eg, Facebook and Twitter) or email. The included HIV/STD facts on this app were not comprehensive and it was not clear how the specific facts were chosen. For example, only 2 facts referred to HIV transmission/acquisition: “HIV is transmitted through the blood” and “According to the Centers for Disease Control and Prevention (CDC), using a condom every time you have sex can lower your risk of HIV infection.” Regarding other sexually transmitted diseases, only human papillomavirus (HPV) was mentioned by name in a factoid that stated that HPV “is the world’s most common sexually transmitted infection.” Although sexuality was listed in the app’s description, the overwhelming majority of facts centered on heterosexuality.

### Apps Tailored for Specific Populations

A small proportion of apps appeared to include or target specific populations. Approximately one-quarter (13/55, 24%) of apps were designed for a particular geographic area: England (Big Night Out [36], It’s Your Shout [33], Protection-Sex [37], Safer Sex [38], Sex Guide [39]); China/Singapore (Action For AIDS [22], My First Time [40]), Denmark (Safesex Guide [41]); Ireland (Sexual Health Guide [27]); Birmingham, Alabama (Birmingham AIDS Outreach [42]); Massachusetts (MASTDinfo [43]), New York City (NYC Condom Finder [44]); Philadelphia, Pennsylvania (iCondom Philly [45]); and San Francisco, California (STD411 [24]).

A minority of apps (n=10) were inclusive of information about anal sex or featured information for lesbian, gay, bisexual, or transgender (LGBT) persons. These apps included Birmingham AIDS Outreach [42], HIV RISK Calculator [46], MASTDinfo [43], Safe Sex Tips [26], Safer Sex [38], Sex Detective [47], Sex Guide [39], Sexual Health Guide [27], STD Risk Calculator Lite [48], and STD411 [24]. Of these, the only tailored apps were MASTDinfo [43], which provided information and testing center locations for men who have sex with men (MSM) in Massachusetts, and STD411 [24] that provided information and resources for gay, bisexual, and transgender men in San Francisco (Figure 5).

**Figure 6:**
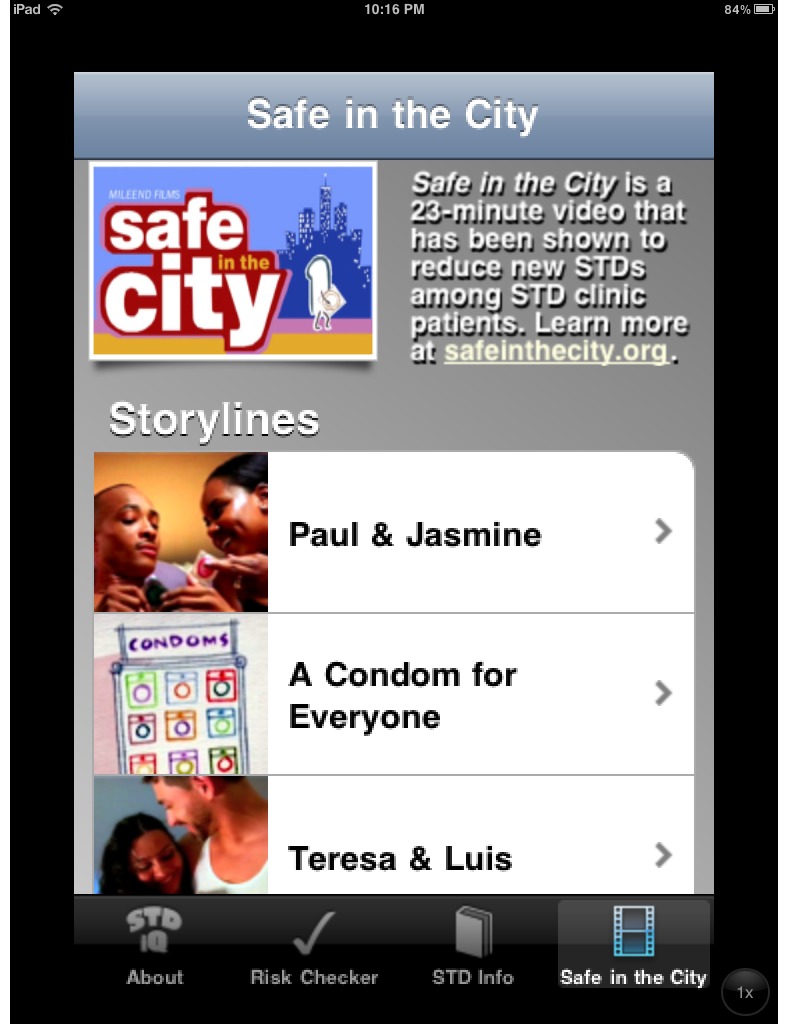
Screenshot of STDiQ app featuring racially/ethnically diverse videos from the Safe in the City STD reduction intervention. iPhone version shown on iPad 2 for clarity. Reproduced with permission from Cornelis A Rietmeijer, MD, PhD, MSPH, University of Colorado, Denver.

**Figure 7:**
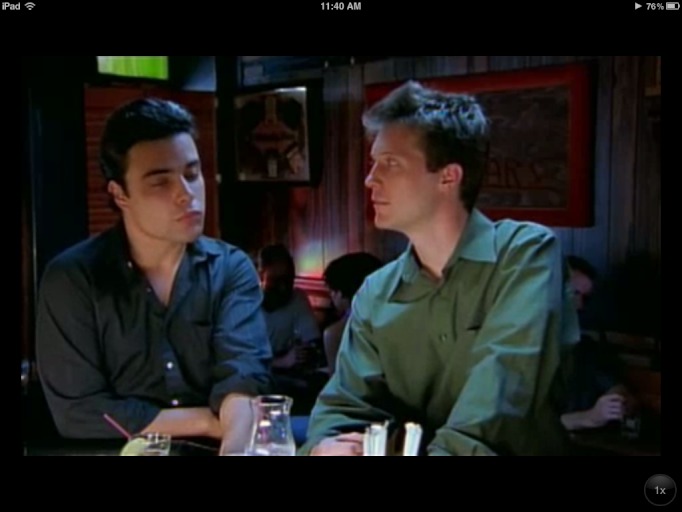
Screenshot of STDiQ app featuring a bar scene from Rueben’s one-night stand in the Safe in the City STD reduction intervention. iPhone version shown on iPad 2 for clarity. Reproduced with permission from Cornelis A Rietmeijer, MD, PhD, MSPH, University of Colorado, Denver.

Nine apps included pictures, text, or videos with persons from racial or ethnic minority groups: AfterSex [49], AIDSinfo HIV/AIDS Glossary [50], Birmingham AIDS Outreach [42], HIV and Your Heart [30], Kenny Condom [51], Men’s Sexual Health [52], Pos or Not [23], STDiQ [25], and TKNO [53]. Among these, STDiQ [25] was tailored for black and Latino men and women. This app features videos from the Safe in the City campaign (Figures 6 and 7), a theory-based intervention found to reduce incident STD cases [54]. Kenny Condom [51] is based on a previously developed character and Web initiative tailored toward young African American men and “the hip-hop generation.”

## Discussion

In our review of Apple iTunes and Android Google Play apps, we found 55 unique apps that fostered HIV/STD prevention and care services. In general, these apps were underutilized and only a few apps received high user ratings. Apps primarily focused on providing disease information, and only 6 apps covered all 4 areas of a basic prevention strategy (knowledge raising, risk reduction, condom promotion, and testing). However, we also found examples of apps that used a tailored and/or interactive approach and apps that illustrated the potential for integrating mobile phone technologies into more comprehensive HIV prevention and care.

There are a number of benefits to using mobile phone apps to provide HIV/STD prevention and care services. Apps offer convenience to both the user and the developer because they provide a flexible way to reach a large audience at an affordable cost. Apps can provide individually tailored and interactive HIV/STD prevention interventions that are constantly accessible and allow the user to seek information while maintaining anonymity. Intervention customization and interactivity have been found to be important for effectiveness in behavior change interventions, including those that are technology based [55]. Well-designed mobile phone apps accommodate self-tailoring and personalization by allowing the user to choose which app features they wish to use and selecting messages and notifications they wish to receive through the app, email, or SMS. Unlike SMS and automated voice messages that are sent at specific times, the interactive functions of mobile phone apps are constantly available, allowing users to engage with the app at their convenience.

The HIV/STD phone apps also capitalize on delivering an intervention in a way that is familiar, desirable, and discrete to at-risk populations including youth, MSM, and racial and ethnic minorities [56]. Within the United States, the black population represents the most active and fastest growing user group of the mobile Internet [57]. And MSM have also been shown to have greater access to and use of cell phone technologies compared to heterosexual populations [58], increasing the likelihood of delivering highly engaging prevention messages to this hard-to-reach population, including those who are not reached through in-person or group interventions [59].

Unfortunately, many of the very features that make apps such a promising platform for delivering HIV/STD prevention and care services appear to be lacking in the currently available HIV/STD-related apps. Based on our review, as of August 1, 2012, less than 0.3% of the more than 29,000 health-related apps available for iPhone and Android consumers [18,19] were dedicated to HIV/STD information and prevention. Furthermore, existing HIV/STD apps have generally failed to attract the attention and positive reviews of target audiences. This is likely because of a combination of inadequate app promotion and failure to create engaging, attractive apps. How can we do better?

Many of the core elements of social marketing offer an appropriate guiding framework for developing health interventions with mobile technologies [60,61]. Limited downloads and low user ratings indicate that most HIV/STD apps have not achieved the ideal social marketing mix of “product, price, place, and promotion.” Social marketing principles, such as audience analysis and segmentation, and the use of formative research during product design would ensure that apps are developed with the user in mind [62]. For example, in focus groups we have held with young, black MSM in North Carolina, more men owned mobile phones that operated on an Android OS rather than an iOS platform (Muessig et al, unpublished). Thus, although a larger overall number of users in this age bracket may own iPhones, those who face a disproportionately high risk of HIV infection (young, black, and MSM) [63] might be better reached with an Android-supported app. Similarly, HIV/STD app developers need detailed information about who does and does not have access to these technologies. For example, general consumer research shows disparities in smartphone ownership by income. However, in our focus groups among young, black MSM, we found consistent smartphone ownership even among men with annual income under US $12,000 (Muessig et al, unpublished).

Relatedly, apps should include content and form that resonates with specific populations and that they want or need to use [64]. For example, an app that features a 3-paragraph narrative description about HIV clinic visits could be transformed into an interactive 10-point checklist of questions and topics that a patient could use to prepare for the appointment. This altered format engages the user and could be designed to allow users to self-tailor by entering their own checklist items.

Social marketing approaches also incorporate systems for process evaluation and iterative feedback. Ideally, app developers should be collecting target audience input throughout the development and usability testing process, as well as regularly updating their apps based on user feedback and advances in the HIV/STD field. Updates are especially critical for apps that provide information about rapidly evolving topics, such as testing locations, support services, and available HIV medications. As with any other intervention, these apps should be evaluated for measures of effectiveness. This requires identifying outcome goals and measurement metrics at the start of app design and development (eg, increased medication adherence, increased condom use, increased HIV testing frequency). Successful technology interventions such as these will require more involved collaborations between developers and public health HIV/STD researchers and practitioners [65].

Market research shows that smartphone owners regularly remove unused apps from their phones, thus design and content must be sufficiently useful and entertaining for consumers to keep them on their phones [66]. One analysis of app retention (as defined by the use of an app in the past 7 days) found a global retention rate of only 14.8% and US rates at a lower 12% [67]. Apps that incorporate more interactive components, such as games, quizzes, and activities, may be able to improve attractiveness and user retention. The categories of “entertainment” and “games” comprise the largest market share of available, active apps (27% to 29%) [19,68] and half of the top 10 most-downloaded apps of 2011 were gaming apps [69]. Game features of health interventions (eg, health-related challenges and rewards, ability to “level up,” and use of avatars) have been shown to promote and sustain healthy behaviors [70-73]. Thus, building in more interactive and gaming features in HIV/STD apps could improve their attractiveness and chances for successful intervention.

In addition to a need for better tailoring, design, and interaction, there were a number of content areas missing from most apps. First, apps for HIV-positive persons generally lacked psychological and emotional support resources and messages about the prevention of STDs and onward HIV transmission. Second, additional tools are needed to promote HIV medication adherence, support clinical monitoring, and facilitate patient-provider relationships. Active technology tools for self-monitoring have facilitated a variety of health improvement programs from increases in physical activity [74] to depression management [75]. Interventions using electronic drug monitoring devices suggest that these tools could also provide benefits for HIV-positive persons in improving medication adherence [76,77]. A few of the apps we identified included these types of tools, and assessments are needed to identify optimal design and efficacy. Additional mechanisms should be tested for providing linkage between the user/patient and their health care team. These features could include remote health coaching, symptom and side effect monitoring, and provision of real-time feedback. Third, a stronger emphasis is needed on interpersonal skills in the context of HIV/STD risk. Very few apps modeled condom negotiation, HIV/STD status disclosure, or sexual decision making in the context of relationships (see STDiQ [25] and Pos or Not [23] for notable exceptions). Finally, there were almost no descriptions of biomedical HIV prevention options, such as earlier initiation of antiretroviral therapy or preexposure and postexposure prophylaxis.

This review has several limitations. Our searches were restricted to the Apple iTunes and Google Play app stores. We selected these phone platforms (iPhone and Android) because they account for over 85% of the global app market [78,79] and, thus, are likely to be representative of the apps available to the majority of smartphone users. However, there may be HIV/STD-related apps available through other platforms that were not captured in our review. In addition, the field of app development is in constant motion with large numbers of new apps being created and other apps being deleted every day. Illustrating the speed of this development, the number of available apps in the 4 leading app stores (Apple iTunes Store, Google Play Store, Windows Phone Store, and Blackberry App World) increased from 989,476 in December 2011 [79] to 1,574,645 in September 2012 [78]. Importantly, our review was not designed to evaluate the effectiveness of identified apps in achieving positive prevention or treatment outcomes. Furthermore, we were not able to assess the user profiles of those who downloaded these apps. Although a growing body of evidence suggests high acceptability and smartphone ownership across diverse users [62,80], as discussed previously, detailed sociodemographic information (eg, user age, education, socioeconomic class, sexual orientation, race/ethnicity, and native language) should critically inform the tailoring and targeting of HIV/STD prevention and care apps.

Providing HIV/STD prevention and care services through mobile phone apps shows great potential for growth, both in improving the acceptability and adoption of existing apps, and creating new HIV/STD apps. Future HIV/STD app development could be informed by the principles of social marketing to build appropriately tailored, interactive apps. As biomedical advances in antiretroviral treatment are bringing the prevention of onward HIV transmission within our reach, we can use the powerful, widespread technologies offered through mobile phone apps to explore behavioral interventions for risk reduction and close gaps in HIV/STD testing, treatment adherence, and retention in care.

## Abbreviations

HIV: human immunodeficiency virus
HPV: human papillomavirus
MSM: men who have sex with men
OS: operating system
SMS: Short Message Service
STD: sexually transmitted disease
